# Intrapleural Migration of an Endobronchial Watanabe Spigot: Persistent Air Leak and Surgical Retrieval

**DOI:** 10.1016/j.atssr.2025.06.022

**Published:** 2025-07-22

**Authors:** Yoshiki Chiba, Masahiro Miyajima, Kazuya Honda, Kazuki Sato, Takeshi Ohyu, Yuki Takahashi, Ryunosuke Maki, Atsushi Watanabe

**Affiliations:** 1 Department of Thoracic Surgery, Sapporo Medical University School of Medicine and Hospital, Sapporo, Hokkaido, Japan

## Abstract

Bronchial occlusion with an endobronchial Watanabe spigot (EWS) is an effective intervention for managing persistent air leak. Severe complications associated with EWS are uncommon. We highlight a rare case of EWS migration into the pleural cavity following bronchial occlusion for persistent air leak in a patient with acute respiratory distress syndrome secondary to influenza pneumonia after blunt chest trauma. The patient underwent successful video-assisted thoracoscopic surgery for EWS retrieval and pulmonary fistula repair, resulting in an uneventful recovery without recurrence of pneumothorax. This case underscores the importance of appropriate spigot selection, anatomic assessment, and vigilant monitoring in structurally compromised lungs.

Persistent air leak (PAL) poses a clinical challenge in patients with underlying pulmonary disease or after trauma. The endobronchial Watanabe spigot (EWS), made of silicone, is a bronchoscopic tool for nonsurgical PAL management.^1^, ^2^, ^3^ Spigot migration into the central airway is relatively common; however, intrapleural migration is exceedingly rare and infrequently reported.^4^ We describe a rare case of intrapleural EWS migration necessitating surgical intervention, with a discussion of possible mechanisms and preventive considerations.

A 60-year-old male patient with no significant past medical history sustained thoracic trauma in a motor vehicle accident. Initial imaging revealed a mild right-sided pneumothorax, sternal fractures, and left-sided rib fractures. On admission, the patient tested positive for influenza A virus. Chest computed tomography (CT) showed bilateral apical bullae without pulmonary contusion or atelectasis.

On day 1 of hospitalization, influenza pneumonia developed. Despite antiviral and antibiotic therapies, intubation was required on day 4. Intravenous administration of high-dose methylprednisolone (1000 mg/d for 3 days) failed to improve the clinical condition. The patient was transferred on day 6 for advanced care.

Post-transfer radiography revealed extensive infiltration shadow and decreased permeability in the left lung field (Figure 1A). Chest CT showed bilateral ground-glass opacities, consolidations, bronchial dilation, and pleural effusion, consistent with acute respiratory distress syndrome secondary to influenza pneumonia complicated by suspected organizing pneumonia. Venovenous extracorporeal membrane oxygenation (VV-ECMO) was initiated on day 8, along with prone positioning. Improvement in gas exchange facilitated ECMO decannulation on day 15, followed by tracheostomy.

**Figure 1:**
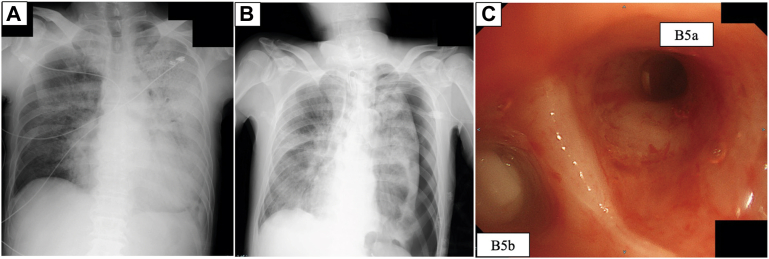
(A) Chest radiograph on hospital day 6 showing extensive infiltration and reduced aeration in the left lung field. (B) Chest radiograph on hospital day 19 showing a left-sided pneumothorax. (C) Bronchoscopic view on hospital day 20 showing endobronchial Watanabe spigots placed in B5a and B5b.

On day 19, a left-sided pneumothorax developed, necessitating chest tube insertion (Figure 1B). A major air leak was observed, and adhesive therapy was avoided because of the risk of aspiration pneumonia. On day 20, less invasive bronchoscopic treatment preceded surgical treatment.

The procedure was performed without fluoroscopy in the emergency unit. Balloon occlusion testing identified the left lingular segment as the source. Seven EWS devices were deployed into B4a, B4b, B5a, and B5b (Figure 1C). Although transient suppression was observed, the leak recurred immediately. On day 21, radiography suggested EWS displacement (Figure 2A, 2B). Subsequent CT confirmed intrapleural spigot migration, with a visible tract from B5a to the visceral pleura, indicating a direct path of extrusion (Figure 2C, 2D).

**Figure 2:**
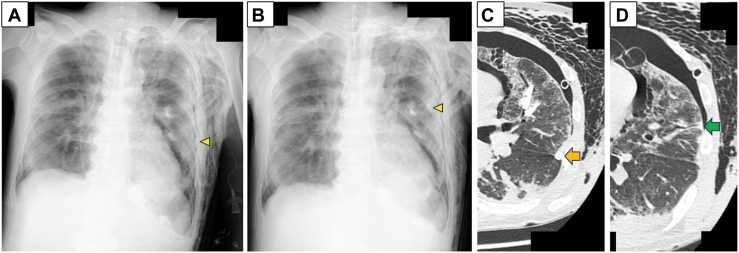
Chest radiographs on hospital days (A) 20 and (B) 21 after bronchoscopic intervention showing endobronchial Watanabe spigot displacement (arrowhead). (C) Chest computed tomography on hospital day 21 showing intrapleural migration of 1 spigot (arrow). (D) Corresponding axial image showing a tract extending from B5a to the visceral pleura (arrow).

On day 24, the patient was evaluated by the thoracic surgery department, and thoracic surgery was planned owing to persistent PAL unresponsive to less invasive treatment. Considering the patient’s compromised respiratory condition, VV-ECMO was reinitiated. The patient was placed in the right lateral decubitus position. Adequate drainage and perfusion were maintained throughout.

Video-assisted thoracic surgery revealed an EWS free in the pleural cavity and a pulmonary fistula at the lingular surface, not associated with a bulla (Figure 3A; Video). The fistula was closed by horizontal mattress sutures reinforced with fibrin glue, polyglycolic acid sheets, and free pericardial fat pledgets. Multiple apical bullae without active leakage were treated prophylactically with fibrin glue and polyglycolic acid sheets. The operative time was 98 minutes, with minimal blood loss. The migrated EWS, originally placed in B5a, was confirmed to be size L. Its tip had been trimmed during preparation (Figure 3B).

**Figure 3:**
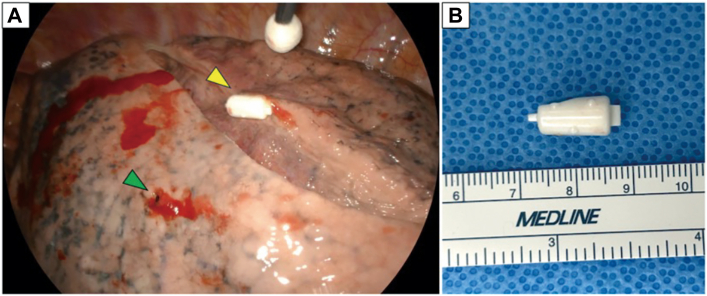
(A) Intraoperative view showing an endobronchial Watanabe spigot freely located within the pleural cavity (yellow arrowhead) and a pulmonary fistula on the lingular surface (green arrowhead). (B) Retrieved endobronchial Watanabe spigot, originally placed in B5a, confirmed to be size L.

The postoperative course was uneventful. VV-ECMO was discontinued on postoperative day 7 and the chest tube was removed on day 11, with no pneumothorax recurrence. The patient was transferred to rehabilitation on day 34. Ventilator weaning was achieved on day 55, and the patient was discharged approximately 4 months after surgery.

## Comment

EWS-based bronchial occlusion serves as a minimally invasive and effective modality for managing PAL, particularly in patients who are unsuitable for surgical repair. Although it is commonly used, complications such as pneumonia, mucous plugging, and spigot migration can occur. A 2010 nationwide retrospective survey conducted by the Japan Society for Respiratory Endoscopy reported 227 EWS bronchial occlusion procedures performed across 86 institutions.^5^ Only 3 complications (2 pneumonia cases and 1 respiratory failure case) were documented, with no reports of intrapleural spigot migration. A case of delayed migration into the pleural space 6 months after EWS placement has been reported in a patient with persistent pneumothorax due to chronic *Aspergillus* infection.^6^ EWS migration through preexisting cavitary lesions has been associated with pulmonary aspergillosis, suggesting that such cavities may serve as potential pathways for device displacement. However, that case generally followed a chronic clinical course before migration. By contrast, this case demonstrated acute EWS migration within 24 hours of placement. Despite the presence of multiple pulmonary cysts, the spigot dislodged through a fistula that developed on the surface of radiologically normal lung parenchyma, unrelated to any cystic structure. This case highlights the potential for EWS migration even without identifiable structural abnormalities, such as cavities or cysts. In patients with compromised pulmonary parenchyma due to acute respiratory distress syndrome, organizing pneumonia, steroid therapy, or posttraumatic fibrosis, spigot dislodgment may occur through apparently intact lung tissue. Clinicians should remain vigilant for early EWS migration in such settings.

Despite appropriate size selection (L-sized spigot for B5a), the patient’s bronchial architecture may have increased susceptibility to extrusion. This highlights the importance of individualized assessment of bronchial wall integrity and consideration of adjunctive fixation methods or enhanced postprocedural imaging, particularly in structurally compromised lungs.

Although it is not routinely recommended, fluoroscopic guidance during EWS placement may help prevent excessive distal deployment and facilitate early detection of dislodgement, particularly in patients with structurally fragile lungs.

In conclusion, we report a rare case of intrapleural migration of an EWS after bronchial occlusion for a PAL. The complication was managed successfully with thoracoscopic spigot retrieval and pulmonary fistula repair through the video-assisted thoracic surgery approach. This case underscores the importance of appropriate spigot sizing, anatomic evaluation, and vigilant monitoring in patients with fragile pulmonary architecture.
